# The Calcineurin-Binding, Activity-Dependent Splice Variant Dynamin1xb Is Highly Enriched in Synapses in Various Regions of the Central Nervous System

**DOI:** 10.3389/fnmol.2017.00230

**Published:** 2017-07-25

**Authors:** Marie-Lisa Eich, Ekta Dembla, Silke Wahl, Mayur Dembla, Karin Schwarz, Frank Schmitz

**Affiliations:** Department of Neuroanatomy, Medical School Homburg/Saar, Institute for Anatomy and Cell Biology, Saarland University Homburg/Saar, Germany

**Keywords:** dynamin1xb, splice variant, synapse, retina, darkness-induced synaptic recruitment of dynamin1xb, calcineurin

## Abstract

In the present study, we generated and characterized a splice site-specific monoclonal antibody that selectively detects the calcineurin-binding dynamin1 splice variant dynamin1xb. Calcineurin is a Ca^2+^-regulated phosphatase that enhances dynamin1 activity and is an important Ca^2+^-sensing mediator of homeostatic synaptic plasticity in neurons. Using this dynamin1xb-specific antibody, we found dynamin1xb highly enriched in synapses of all analyzed brain regions. In photoreceptor ribbon synapses, dynamin1xb was enriched in close vicinity to the synaptic ribbon in a manner indicative of a peri-active zone immunolabeling. Interestingly, in dark-adapted mice we observed an enhanced and selective enrichment of dynamin1xb in both synaptic layers of the retina in comparison to light-adapted mice. This could be due to an illumination-dependent recruitment of dynamin1xb to retinal synapses and/or due to a darkness-induced increase of dynamin1xb biosynthesis. These latter findings indicate that dynamin1xb is part of a versatile and highly adjustable, activity-regulated endocytic synaptic machinery.

## Introduction

Dynamins are large GTP-binding mechanoenzymes that are essential for various types of membrane retrieval and vesicle fission at the plasma membrane (for review, see McMahon and Boucrot, 2011; Schmid and Frolov, 2011; Ferguson and De Camilli, 2012; Kirchhausen et al., 2014; Kononenko and Haucke, 2015; Soykan et al., 2016). In addition, dynamin is also involved in membrane fission at distinct endomembrane systems, e.g., the Golgi apparatus and endosomes (Cao et al., 1998; Jones et al., 1998; McNiven et al., 2000; Praefcke and McMahon, 2004; Schulze et al., 2013; Kononenko et al., 2014; Watanabe et al., 2014; Arlt et al., 2015; Soykan et al., 2016). The trafficking processes in which dynamins are involved are functionally diverse and not only include constitutive but also activity-regulated membrane trafficking pathways (for review, see Ferguson and De Camilli, 2012; Watanabe and Boucrot, 2017). Dynamin also interacts with components of the actin cytoskeleton (for review, see Ferguson and De Camilli, 2012; Sever et al., 2013; Wu L.-G. et al., 2014; Kononenko and Haucke, 2015; Soykan et al., 2016). This interaction with the actin cytoskeleton is important for endocytic membrane retrieval and vesicle replenishment (e.g., Hayashida et al., 2015; Wen et al., 2016; Wu et al., 2016; Soykan et al., 2017; for review, see Rizzoli, 2014; Kononenko and Haucke, 2015; Soykan et al., 2016; Herrero-Garcia and O’Bryan, 2017). Besides its well-known essential role in vesicle fission, dynamin has been proposed to be involved in certain aspects of membrane fusion, e.g., fusion pore stabilization and expansion (Peters et al., 2004; Anantharam et al., 2011, 2012; Samasilp et al., 2012, 2014; Alpadi et al., 2013; González-Jamett et al., 2013; Kulkarni et al., 2014; Jackson et al., 2015; Zhao et al., 2016; for review, see Antonny, 2004; Sever et al., 2013; Quan and Robinson, 2014; Ren et al., 2016). In mammalian cells, dynamin-dependent tasks have to be accomplished by the gene products of three dynamin genes, dynamin1-3 (Cook et al., 1996; Urrutia et al., 1997; for review, see Ferguson and De Camilli, 2012). Dynamin-1 is brain-specific and expressed at high levels in neurons whereas dynamin-2 is ubiquitously expressed. Dynamin-3 is preferentially expressed in the brain (at much lower amounts than dynamin-1) but is also present in testis and lung (for review, see Ferguson and De Camilli, 2012).

Dynamin proteins possess a well-characterized protein domain structure (Chappie et al., 2011; Faelber et al., 2011; for review, see Schmid and Frolov, 2011; Ferguson and De Camilli, 2012). These domains include an aminoterminal GTPase domain (G-domain), a central lipid-binding pleckstrin homology (PH)-domain, a GTPase-effector domain (GED) that is part of the bipartite stalk region of dynamin as well as a carboxyterminal proline-rich domain (PRD). The PRD of dynamin mediates binding of various SH3-domain-containing dynamin-interacting proteins. The SH3-domains of different BAR domain-containing proteins, e.g., syndapin, amphiphysin and endophilin, bind to the PRD of dynamin at distinct but overlapping regions (Anggono and Robinson, 2007; Clayton et al., 2009; Xue et al., 2011; Luo et al., 2016; for review, see Cousin and Robinson, 2001; Clayton and Cousin, 2009; Ferguson and De Camilli, 2012; Cousin, 2015).

Dynamin-1, a member of the dephosphin family of proteins (Cousin and Robinson, 2001), is a phosphoprotein with two major phosphorylation sites in the PRD (at serine S774 and S778 in rat dynamin-1; Anggono et al., 2006; Anggono and Robinson, 2007). At rest, these serines are constitutively phosphorylated. Interaction between dynamin-1 and syndapin requires dephosphorylation of S774/S778 whereas interaction of dynamin-1 with amphiphysin is not inhibited by phosphorylation of these sites (Clayton et al., 2008, 2009, 2010; for review, Clayton and Cousin, 2009). The phosphorylation-dependent dynamin1-syndapin interaction is important for enhancing activity-dependent endocytosis, e.g., via bulk endocytosis (Clayton et al., 2008, 2009, 2010; Clayton and Cousin, 2009) and also has been proposed to be involved in the control of fusion pore expansion (Anantharam et al., 2011, 2012; Samasilp et al., 2012, 2014; González-Jamett et al., 2013; Trouillon and Ewing, 2013; Jackson et al., 2015; Trexler et al., 2016; for review, see Sever et al., 2013; Quan and Robinson, 2014; Ren et al., 2016). De-phosphorylation of dynamin-1 is mediated by the Ca^2+^/calmodulin-binding phosphatase calcineurin (Clayton et al., 2008, 2009, 2010; Clayton and Cousin, 2009; Wu X.-S. et al., 2014).

Various splice variants are produced from neuronal dynamin-1 at different splice sites within the dynamin molecule (Cao et al., 1998; McNiven et al., 2000; Ferguson and De Camilli, 2012). At the very carboxyterminus, two major splice sites of dynamin1 are generated, i.e., a longer dynamin1xa splice variant and a shorter dynamin1xb splice variant (Bodmer et al., 2011; Xue et al., 2011). Dynamin1xb is a particularly interesting dynamin-1 splice variant because it contains a docking site for calcineurin (Bodmer et al., 2011; Xue et al., 2011). The calcineurin docking site makes dynamin1xb very well suited to mediate activity-dependent changes, e.g., in response to elevated synaptic activity, that is associated with elevated cytosolic Ca^2+^ (e.g., Marks and McMahon, 1998; for review, see Kononenko and Haucke, 2015). Furthermore, calcineurin is a central mediator of homeostatic synaptic plasticity (Arendt et al., 2015). Therefore, we studied the distribution of dynamin1xb in various regions of the mouse brain by using a monoclonal antibody that selectively detects the dynamin1xb splice variant of dynamin1. For the morphological analyses, we selected brain regions with a particularly clear and highly ordered cellular organization like the retina and cerebellum in which synaptic layers with well characterized synapses can be readily discriminated from non-synaptic layers. We also included the retina in our analyses because the retina allows high resolution analyses of synapses and displays particularly prominent, activity-/illumination-dependent changes of synaptic activity.

## Materials and Methods

### Animals

Experiments were performed on the described tissues of C57Bl/6J mice of both sexes. Animal care and all experimental procedures were reviewed and approved by the animal welfare and ethics committee of the Saarland University. Mice were kept under standard light/dark cycle and supported with standard food and water *ad libitum*.

### Primary Antibodies

#### Anti-dynamin1xb

The monoclonal antibody against dynamin1xb was generated against the carboxyterminal peptide stretch of dynamin1xb that serves as a docking site for calcineurin (Bodmer et al., 2011; Xue et al., 2011). Monoclonal antibody was raised against the carboxyterminal 12 amino acids (aa; aa840-851; PPGVPRITISDP) of rat dynamin1xb (NP_542420). An additional cysteine at the N-terminus of the peptide served to conjugate the peptide to bovine serum albumin (BSA) prior to immunization. The last seven aa residues (RITISDP) are present only in dynamin1xb but not in dynamin1xa and represent a binding site for the catalytic domain of calcineurin (Bodmer et al., 2011; Xue et al., 2011). Immunization of mice, fusion of spleen cells and selection of ELISA-positive hybridoma clones was done by Absea Biotechnology (Beijing, China) using standard procedures. The hybridoma cell culture supernatant 1E10 (IgG2b immunoglobulin subtype) was used for immunofluorescence (IF) microscopy in a 1:300 dilution (concentration of the primary monoclonal antibody: ≈2.7 μg/ml), for western blotting in a 1:1000 dilution concentration of the primary monoclonal antibody: ≈0.8 μg/ml.

#### Anti-RIBEYE

Polyclonal rabbit antibody (U2656) against RIBEYE(B)-domain (Schmitz et al., 2000). The antibody was used for IF microscopy in a 1:1000 dilution.

#### Anti-RIM1/2

Polyclonal rabbit antibody against RIM1/2 (Schoch et al., 2006; Anjum et al., 2014). This antibody was used for IF microscopy in a 1:250 dilution.

#### Anti-Synaptotagmin-1

Polyclonal rabbit antibody against synaptotagmin-1 (V216, Perin et al., 1990; Pang et al., 2006; Bacaj et al., 2015). This antibody was used for IF microscopy in a 1:250 dilution.

#### Anti-pan-SV2

Mouse monoclonal antibody against the synaptic vesicle protein 2 (SV2; Buckley and Kelly, 1985). Cell culture supernatant was obtained from the Developmental Studies Hybridoma Bank (DSHB), University of Iowa. The cell culture supernatant was used at a 1:50 dilution for IF microscopy.

#### Anti-β-tubulin

Rabbit polyclonal antibody against aa210-aa444 of human β-tubulin (Santa Cruz; #H-235, sc-9104); used for IF microscopy in a 1:150 dilution.

#### Anti-Actin

Mouse monoclonal antibody against actin (clone C4, Millipore MAB1501), used for western blotting in a 1:5000 dilution.

### Secondary Antibodies

#### Secondary Antibodies for Immunofluorescence Microscopy and Western Blotting

Goat anti-mouse immunoglobulins, conjugated to horseradish peroxidase (Sigma, A3673); used for western blotting in a 1:10,000 dilution. Chicken anti-mouse immunoglobulins conjugated to Alexa488 (Invitrogen; #A21200), used for IF microscopy in a 1:10,000 dilution. Donkey anti-rabbit immunoglobulins conjugated to Alexa568 (Invitrogen; #A10042), used for IF microscopy in a 1:10,000 dilution. Donkey anti-mouse immunoglobulins conjugated to Alexa568 (Invitrogen; #A10037), used for IF microscopy in a 1:10,000 dilution. Chicken anti-rabbit immunoglobulins conjugated to Alexa488 (Invitrogen; #A21441), used for IF microscopy in a 1:10,000 dilution. Monovalent Fab fragments rabbit anti-mouse (unconjugated; Fab rabbit anti-mouse IgG (H&L); Rockland Immunochemicals, #810-4102 via Biomol GmbH, Hamburg, Germany), used for IF microscopy in a 1:50 dilution.

### Synthetic Peptides for Dot Blot Experiments

Peptides for dot blot experiments were synthesized from Proteogenix (Illkirch, France) and Scilight Biotechnology LLC (Beijing, China). The following peptides were synthesized: (1) PPGVPRITISDP (12mer; “PP12” peptide); (2) PPGVP (5mer; “PP5” peptide); and (3) RITISDP (7mer; “RP7” peptide).

### Embedding of Tissue for Immunofluorescence Microscopy

Tissue embedding was done exactly as previously described (Wahl et al., 2013, 2016; Dembla et al., 2014). For rapid freezing, small tissue blocks (about 1 mm^3^) in volume were dissected and plunge-frozen, as previously described (Wahl et al., 2013, 2016; Dembla et al., 2014).

### Immunolabeling on Semi-Thin Sections

Immunolabeling was performed on 0.5 μm-thin or 1.5 μm-thin semi-thin sections, as indicated in the respective experiments, after resin removal exactly as previously described (Wahl et al., 2013, 2016; Dembla et al., 2014). From the immunolabeled sections, images were acquired either with a Zeiss epifluorescence microscope setup (Axiovert200M) equipped with the respective filter blocks or with a Nikon A1R confocal microscope, as indicated in the respective experiments. In most double immunolabeling analyses, the two primary antibodies were generated in different animal species (i.e., mouse and rabbit, respectively). In these cases, the binding of the primary antibodies could be readily visualized by using secondary antibodies that are directed against the species-specific portion of the respective primary antibodies, as previously described (Wahl et al., 2013, 2016; Dembla et al., 2014). Incubation with the two different primary antibodies as well as incubation with the two different secondary antibodies was done simultaneously. Controls were done by omitting the primary antibodies and by using only the secondary antibodies or by using irrelevant primary antibodies. In one set of double-immunolabeling experiments (Figure 5C), the two primary antibodies were from the same species, i.e., from mouse (double–immunolabeling experiments with anti-panSV2 and anti-dynamin1xb). In order to discriminate the binding of two different monoclonal primary antibodies that were generated in the same species, the procedure of Lewis-Carl et al. (1993) was employed for the immunolabeling of the semi-thin sections. For this purpose, semi-thin sections were first incubated with anti-dynamin1xb mouse monoclonal antibody (overnight (ON), 4°C). After several washes with PBS, the binding of the primary antibody was detected with chicken anti-mouse secondary antibody conjugated to Alex488 (1 h, RT). Residual binding sites of tissue-bound mouse primary antibody were blocked using rabbit polyclonal, monovalent Fab fragments anti-mouse IgG (1:50 dilution; 3 h, RT). Then, after several washes with PBS, the second mouse primary antibody (anti-panSV2) was added (1:50 dilution; ON, 4°C). The binding of this mouse primary antibody was subsequently detected by donkey anti-mouse secondary antibody conjugated to Alexa568 (1:1000 dilution, 1 h, RT). Controls were done by performing the described immunolabeling procedure but with one (of the two) primary antibodies omitted to judge on the specificity of the immunosignals and to check for possible cross-talks between the two different immunosignals. No crosstalk signal was observed in these control incubations (see also Figure 6).

**Figure 1:**
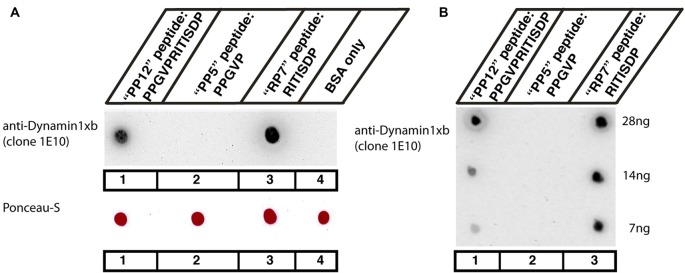
**(A,B)** Dot blot analyses of the indicated peptides for their reactivity with the dynamin1xb antibody. Three different dynamin1xb peptides with the indicated amino acid (aa) sequence (“PP12”: PPGVPRITISDP, “PP5”: PPGVP, “RP7”: RITISDP) cross-linked to bovine serum albumin (BSA) were spotted on nitrocellulose membrane. BSA only, i.e., BSA with no peptides cross-linked to it, served as a further control in **(A)**. As expected, the dynamin1xb antibody strongly reacted with the 12mer dynamin1xb peptide (“PP12”) that was used for immunization (spot #1). The antibody also strongly reacted with the dynamin1xb specific carboxyterminal peptide “RP7” that is specific for dynamin1xb (spot #3) but not with the 5mer peptide “PP5” that is common to both dynamin1xa and dynamin1xb (spot #2). The dynamin1xb antibody also did not react with BSA alone (spot #4). Fifty microgram of cross-linked peptide were spotted in **(A)**. Despite the high amount of the peptides spotted in **(A)**, the dynamin 1xb selectively reacts with RP7 (spot #3), the dynamin1xb-specific peptide region but not with “PP5” (spot #2). **(B)** Similar dot blot analyses as also shown in **(A)** but with less conjugated peptide spotted to the nitrocellulose membrane. The antibody against dynamin1xb selectively detects the “RP7” peptide of dynamin1xb down to an amount of 7 ng. The sensitivity towards the “RP7” peptide appears to be even higher than the sensitivity towards the “PP12” peptide as judged by the immunostaining intensity of the respective peptide spots with the dynamin1xb antibody.

**Figure 2:**
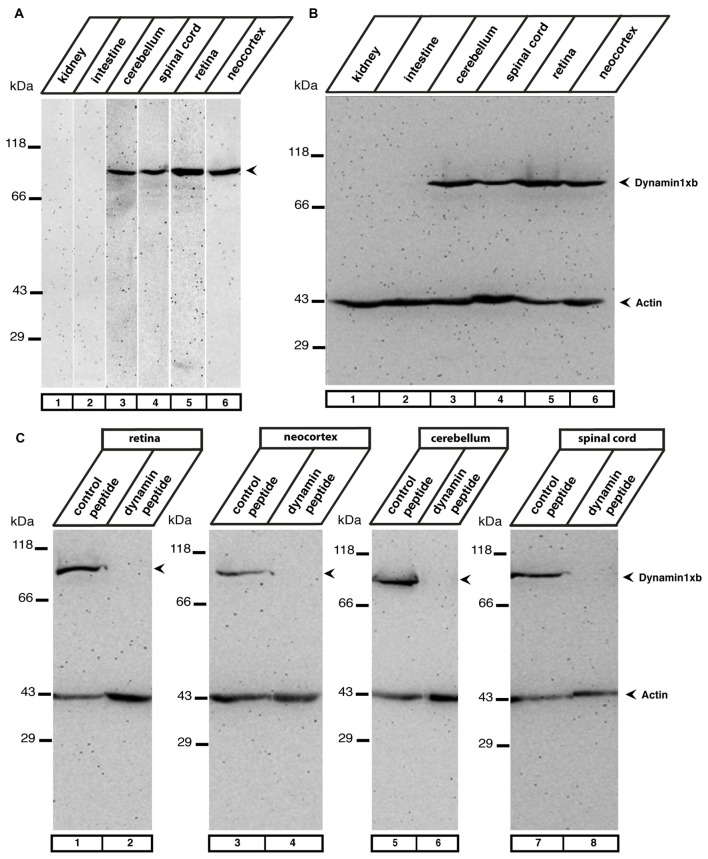
**(A)** The indicated mouse tissues were tested for the presence of dynamin1xb with the characterized monoclonal antibody 1E10 by western blot analyses. We observed a single band at the expected running position of ≈100 kDa in neuronal tissues (cerebellum, retina, spinal cord, neocortex, lanes 3–6) but not in extra-neuronal tissues (kidney, intestine; lanes 1,2). In **(B)** the same tissue extracts as in **(A)** were loaded and tested subsequently with the dynamin1xb antibody and a monoclonal antibody against actin, that served as a loading control. Similar as in **(A)**, dynamin1xb was only present in the neuronal tissues whereas the actin immunosignal was present in all tissues at ≈43 kDa. **(C)** The specificity of the dynamin1xb immunolabeling was further corroborated by blocking experiments. The ≈100 kDa band disappeared if the dynamin1xb was pre-absorbed with the dynamin1xb “PP12” peptide (lanes 2,4,6,8) but was unaffected if the antibody was pre-absorbed with an unrelated peptide (lanes 1,3,5,7). Immunodetection of actin at ≈43 kDa served as a loading control.

**Figure 3:**
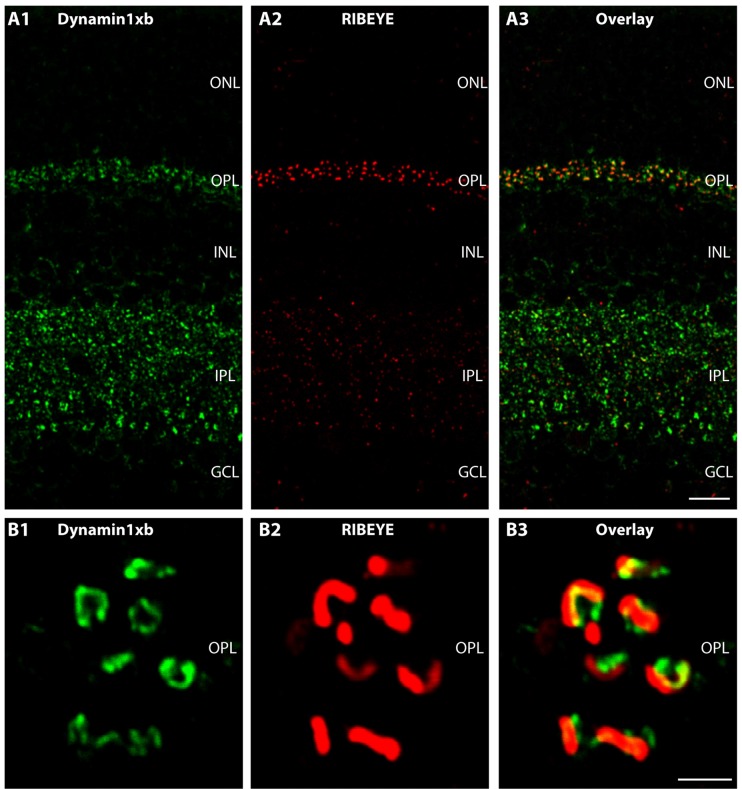
Semi-thin (0.5 μm-thin) sections of the mouse retina immunolabeled with the monoclonal antibody against dynamin1xb. Very predominantly, the synaptic layers of the retina, the OPL and the IPL were immunolabeled by the dynamin1xb antibody. The synaptic layers were visualized by double-immunolabeling with rabbit polyclonal antibodies against RIBEYE (U2656; Schmitz et al., 2000), a major component of synaptic ribbons. In the synaptic layers, the dynamin1xb signal was discrete and displayed a spot-like distribution at low magnification **(A)**. High-resolution confocal microscopy **(B)** of the dynamin1xb immunosignals in the OPL where photoreceptor ribbon synapses are located revealed that the dynamin1xb immunolabeling is highly enriched in close vicinity to the synaptic ribbon. The labeling pattern is very similar to the previously observed immunolabeling pattern with a dynamin antibody that did not discriminate between distinct splice variants, e.g., dynamin1xa and dynamin1xb (Wahl et al., 2013). Figure 3 was obtained by confocal microscopy. Abbreviations: ONL, outer nuclear layer; OPL, outer plexiform layer; INL, inner nuclear layer; IPL, inner plexiform layer; GCL, ganglion cell layer. Scale bars: 20 μm **(A)**; 1 μm **(B)**.

**Figure 4:**
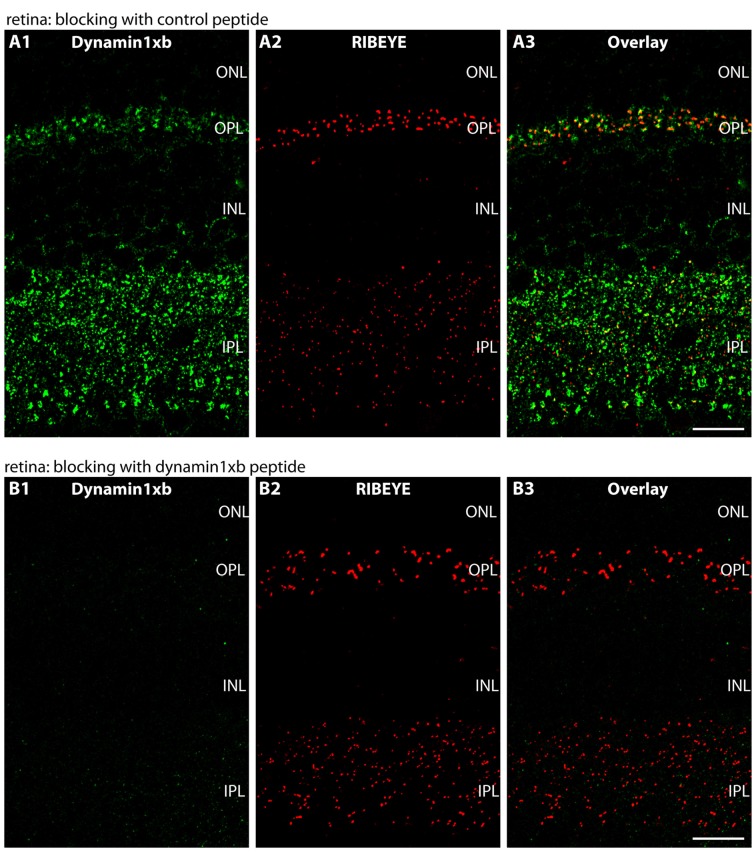
Semi-thin (0.5 μm-thin) sections of the mouse retina immunolabeled with the monoclonal dynamin1xb antibody that was pre-absorbed either with a control peptide **(A)** or with the dynamin1xb peptide “PP12” against which the monoclonal antibody was raised **(B)**. The strong dynamin1xb immunosignal is completely absent if the antibody against dynamin1xb is pre-absorbed with “PP12” whereas the synaptic immunolabel is completely unaffected if a control peptide was used. The RIBEYE immunolabeling was unaffected by both of these treatments. Figure 4 was obtained by confocal microscopy. Abbreviations: ONL, outer nuclear layer; OPL, outer plexiform layer; INL, inner nuclear layer; IPL, inner plexiform layer. Scale bars: 20 μm.

**Figure 5:**
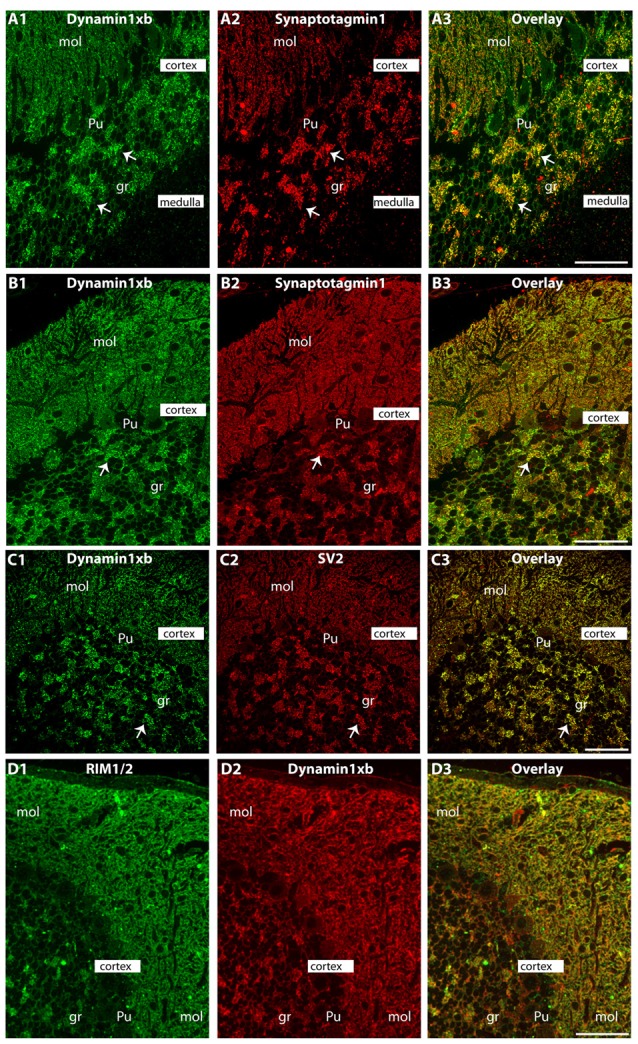
Semi-thin (0.5 μm-thin) sections of the mouse cerebellum double-immunolabeled with the monoclonal dynamin1xb antibody and the indicated other primary antibodies. The other primary antibodies against synaptotagmin1 **(A,B)**, synaptic vesicle protein 2 (SV2; **C**) and RIM1/2 **(D)** were applied to label the synapses in order to better relate the dynamin1xb immunosignals to the synaptic regions. We observed a strong dynamin1xb immunosignal in the cerebellar cortex whereas the cerebellar medulla (white matter) that contains predominantly fiber tracts (but no synapses) was not immunolabeled. In the cerebellar cortex, dynamin1xb was highly enriched in the synaptic regions, i.e., the molecular layer (mol) of the cerebellar cortex and the giant synapses in the granule cell layer (arrows) of the cerebellar cortex. No significant dynamin1xb immunosignal was observed in the medulla of the cerebellum that predominantly contains axonal fiber tracts. **(A,B,D)** was obtained by epifluorescence microscopy; **(C)** was obtained by confocal microscopy. Abbreviations: mol, molecular layer; Pu, Purkinje cell layer; gr, granule cell layer. Scale bars: 50 μm **(A–D)**.

**Figure 6:**
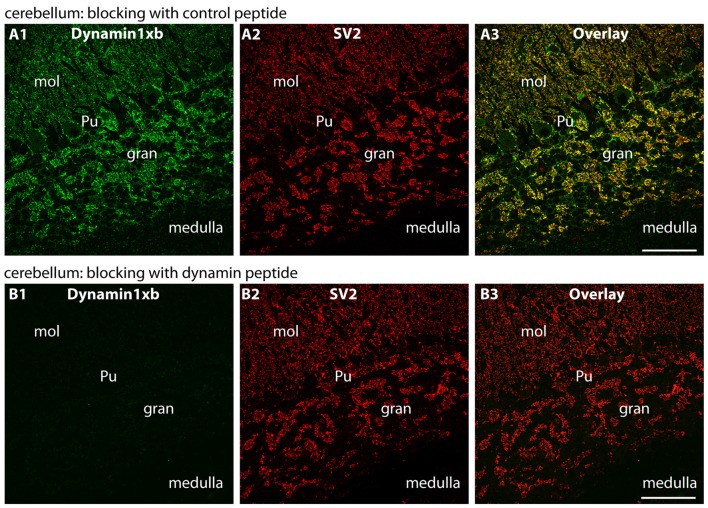
Semi-thin (0.5 μm-thin) sections of the mouse cerebellum immunolabeled with the monoclonal dynamin1xb antibody that was pre-absorbed either with a control peptide **(A)** or with the dynamin1xb peptide “PP12” against which the monoclonal antibody was raised **(B)**. The strong dynamin1xb immunosignal in the synaptic regions of the cerebellar cortex was completely absent if the antibody against dynamin1xb was pre-absorbed with “PP12” whereas the synaptic dynamin1xb immunolabel is completely unaffected if a control peptide was used. The SV2 control immunolabeling was completely unaffected by both of these treatments. Figure 6 was obtained by confocal microscopy. Abbreviations: mol, molecular layer; Pu, Purkinje cell layer; gr, granule cell layer. Scale bars: 30 μm.

### Light- and Dark-Adaptation of Mice

For light- and dark-adaptation experiments, 10 weeks old C57Bl/6J mice were used. Mice were placed either in light (at ≥30 cd/m^2^) or complete darkness (<0.008 cd/m^2^) for 4.5 h. Keeping the animals either in the dark or in the light was done simultaneously, i.e., at the same time of the day, in order to minimize a possible influence from circadian rhythms on the experiments. The experiments were performed between noon and 4:30 pm (exposure started at noon, ended at 4:30 pm). Light intensity was measured with a X9-1 Optometer equipped with a LDM-9901-04 luminance detector (Gigahertz-Optik; Germany). Mice were euthanized by cervical dislocation after isoflurane anesthesia. Isolation of the eyes from light-adapted mice was done as previously described (Grabner et al., 2015). Removal of the eyes from dark-adapted mice (including euthanization) was done under infrared light in complete darkness. After removal, eyes were immediately placed in physiological saline solution with low Ca^2+^ concentration (“LCS” solution, Wahl et al., 2016) on a dissecting microscope stage equipped with infrared illumination and an infrared detection system. For this purpose, the standard binocular setup of the dissecting microscope (Wild M3B, Heerbrugg, Switzerland) was replaced with an infrared viewing system (FJW optical system, Cat. No. 84499A). An infrared illuminator (Conrad Electronics, Model no. CCD-328H) was placed close to the dissecting stage together with an infrared flashlight (NITECORE, Chameleon series CI6, 850 nm infrared light, 1500 mW) that was mounted to the dissecting stage to provide a good infrared illumination. Using this setup to work in complete darkness, the isolated eye was punctured with a 20G needle in the equatorial plane and the anterior part of eye including the lens and the vitreous body were removed after making a circular cut with dissecting scissors. Next, the posterior eyecups with the attached retinas were cryo-preserved in liquid nitrogen-cooled isopentane as previously described (Schmitz et al., 2000; Wahl et al., 2013, 2016; Dembla et al., 2014) in complete darkness with the help of the infrared illumination. The further processing of the frozen samples for IF microscopy was done as previously described (Wahl et al., 2013, 2016; Dembla et al., 2014).

### Quantification of Immunofluorescence Signals

For quantitative analysis, images were acquired using NIS elements software with an A1R Confocal microscope (Nikon), identical conditions were maintained for light and dark adapted retinal immunolabeling using the “re-use” settings option. For quantification images were analyzed using Fiji ImageJ 1.5h software (NIH) and the fluorescence intensity was determined as integrated density. Values were normalized and light values were set to 100%. All the analysis was performed without manipulating any parameters in individual channels as previously described (Wahl et al., 2016). Analyses were done blindly. The areas of outer plexiform layer (OPL) and inner plexiform layer (IPL) were selected by considering dynamin1 labeling as reference because dynamin1 is already known as peri-active zone marker (Wahl et al., 2013). Integrated density was measured for these areas. Then the identical region-of-interests (ROIs) were used to analyze the integrated density for β-tubulin. Statistical analysis was performed using Mann-Whitney rank sum test (as the data were not normally distributed) with the help of Sigma plot software.

### Cross-Linking of Peptides to Bovine Serum Albumin (BSA); Dot Blot Experiments

Equal amounts of the dynamin peptides were cross-linked to BSA by incubation with glutaraldehyde (final concentration 1% in PBS) for 1 h on ice. Afterwards, NaBH_4_ was added (0.1% f.c.) for 15 min at room temperature to block unreacted aldehyde groups. Fifty microgram of cross-linked peptide were spotted to nitrocellulose as indicated in Figure 1A. An equal amount of unconjugated BSA served as negative control to test for possible unspecific binding. Conjugated peptides were spotted on the nitrocellulose membrane in a volume of 5 μl. Samples were allowed to dry for ≈15 min. Afterwards, the nitrocellulose membrane was stained with Ponceau-S and documented. After destaining in PBS, the nitrocellulose membrane was treated with 5% skim milk powder in PBS (blocking buffer) for 60 min at RT to block unspecific protein binding sites of the nitrocellulose membrane. Afterwards, the dot blots were incubated with the dynamin1xb antibody in a 1:1000 dilution in blocking solution (ON, 4°C). After several washes with PBS, binding of the primary antibody was detected by goat anti-mouse secondary antibody conjugated to peroxidase (1:10,000 dilution in blocking buffer; 1 h, RT) and analyzed by enhanced chemiluminescence as previously described (Wahl et al., 2013, 2016; Dembla et al., 2014). In Figure 1B, the peptide was cross-linked with sulfosuccinimidyl 4-[N-maleimidomethyl]cyclohexane-1-carboxylate (Sulfo-SMCC;Thermo Scientific; CAS#: 92921-24-9) in amine-free 5 mM Tris-(2-carboxyethyl)-phosphine buffer (TCEP; Thermo Scientific; product number: 77720), according to the manufacturer’s instructions.

### Miscellaneous Methods

SDS-PAGE and western blotting experiments were performed as previously described (Schmitz et al., 2000; Wahl et al., 2013, 2016; Dembla et al., 2014).

## Results

The monoclonal antibody used in the present study was generated against the 12 carboxyterminal aa of dynamin1xb (PPGVPRITISDP; aa840-aa851 of rat dynamin1; “PP12” peptide). From this stretch of 12mer peptide, the carboxyterminal 7mer peptide (RITISDP; “RP7” peptide) is specific to dynamin1xb while the aminoterminal 5mer peptide (PPGVP; “PP5”) is also contained in dynamin1xa (Bodmer et al., 2011; Xue et al., 2011). In order to determine which region of the 12mer peptide the dynamin1xb monoclonal antibody (clone 1E10) detects, we performed dot blot experiments with the indicated peptides “PP12”, “RP7” and “PP5”. In the dot blot experiments, all peptides (“PP5”, “RP7” and “PP12”) were conjugated to BSA and tested for their reactivity with the generated monoclonal antibody. These dot blot analyses demonstrated that the antibody clone 1E10 only detected PP12 and RP7 but not PP5 even at very high concentrations (Figure 1A). Therefore, the monoclonal antibody from the hybridoma clone 1E10 is specific for the last carboxyterminal aa (RP7) and thus specific for the dynamin1 splice variant dynamin1xb. The monoclonal antibody secreted by the hybridoma clone 1E10 is denoted as dynamin1xb antibody in the following text. The dynamin1xb antigen was detected by the monoclonal antibody in a sensitive manner. Small amounts of “RP7” peptide as low as 7 ng were specifically detected by the monoclonal antibody (Figure 1B).

In western blot analyses, the antibody against dynamin1xb detected a single band at the expected running position for dynamin1 at ≈100 kDa in the neuronal tissues tested (retina, cerebellum, spinal cord, neocortex). The protein was absent in non-neuronal tissue, i.e., kidney and intestine (Figure 2A). Immunolabeling of the western blots with anti-actin served as loading control (Figure 2B). The specificity of the 100 kDa dynamin band detected by the dynamin1xb antibody was further confirmed by pre-absorption experiments. Pre-absorption of the monoclonal dynamin1xb antibody with the specific peptide antigen “PP12” completely blocked immunolabeling of the 100 kDa band while pre-absorption with an unrelated peptide had no effect (Figure 2C).

We used the monoclonal antibody specific for dynamin1xb to analyze the distribution of this dynamin1 splice variant in different parts of the central nervous system. In the retina, we found dynamin1xb highly enriched in the synaptic layers, the OPL and the IPL (Figures 3, 4). In the OPL, photoreceptor ribbon synapses are located that possess a single large active zone with particularly large synaptic ribbons. Therefore, the OPL is very well suitable for high resolution immunolabeling analyses (Wahl et al., 2013, 2016; Dembla et al., 2014). Higher resolution confocal immunolabeling analyses of the distribution of dynamin1xb in the OPL revealed that dynamin1xb is localized in a ring-like manner in close vicinity to the synaptic ribbon (Figure 3). This immunolabeling is very reminiscent to the general dynamin1 immunolabeling that was previously shown to originate predominantly from the peri-active zone at the ultrastructural level. Unfortunately, the dynamin1xb antibody did not work at the ultrastructural level both with pre- and postembedding techniques so that the precise ultrastructural distribution could not be determined. The observed dynamin1xb immunosignal in the synaptic layers of the retina is specific because it could be blocked by pre-absorption of the antibody with the specific dynamin peptide (Figure 4B) but not by pre-absorption with an unrelated control peptide (Figure 4A). The immunolabeling of another protein, i.e., RIBEYE, the main component of synaptic ribbons (Schmitz et al., 2000; Maxeiner et al., 2016), remained unchanged under both of these conditions emphasizing the specificity of the pre-absorption experiments.

Similarly, also in the cerebellum we observed a synaptic enrichment of dynamin1xb as judged by immunolabeling with the monoclonal antibody. The dynamin1xb antibody strongly immunolabeled the molecular layer of the cerebellum in which parallel fibers of granule cells form synaptic contacts onto the dendrites of Purkinje cells (Figure 5). Additionally, the giant synapses in the granule layer of the cerebellum displayed a strong immunoreactivity (Figure 5). Again, pre-absorption experiments further documented the specificity of the immunolabeling results (Figure 6). Pre-absorption of the antibody with the dynamin1xb “PP12” peptide abolished dynamin1xb immunolabeling (Figure 6B1) while control immunolabelings (anti-panSV2; Figure 6B2) were unaffected (Figure 6B). In contrast, pre-absorption of the antibody with an unrelated peptide had no influence on the dynamin1xb immunosignal (Figure 6A1).

**Figure 7:**
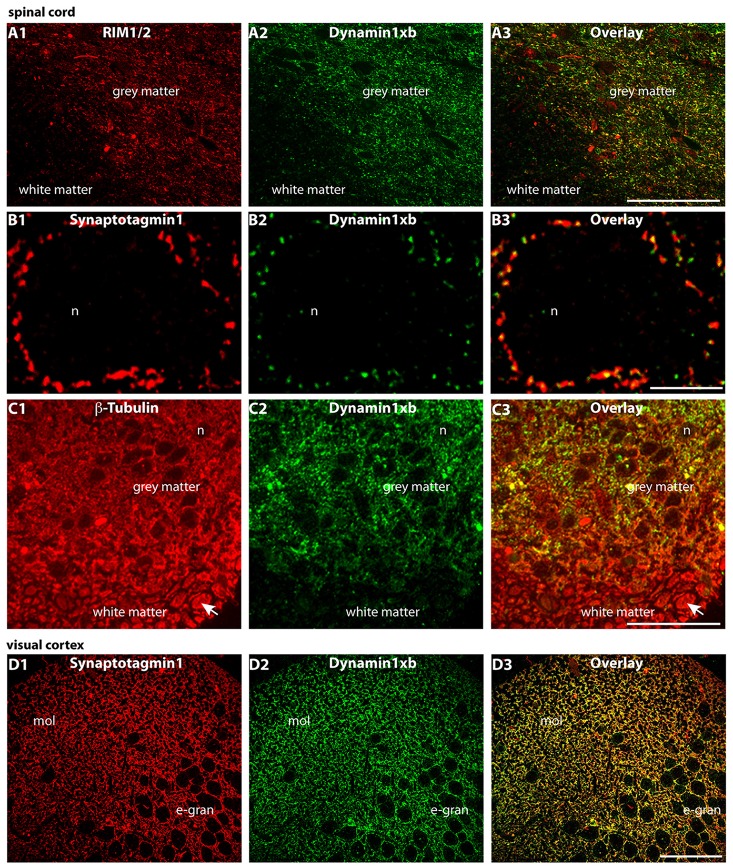
Semi-thin (0.5 μm-thin) sections of the mouse spinal cord **(A–C)** and the mouse visual cortex **(D)** double-immunolabeled with the monoclonal antibody against dynamin1xb and the indicated other primary antibodies. The rabbit polyclonal antibodies against synaptotagmin1 **(B,D)** and RIM1/2 **(A)** were applied to label the synapses in the spinal cord. Immunolabeling with rabbit polyclonal antibodies against β-tubulin was used to also visualize the neuronal axons in the white matter of the spinal cord **(C)**. We observed a strong dynamin1xb immunosignal in the gray matter of the spinal cord whereas the white matter that contains many axons (but virtually no synapses) was largely unlabeled by the dynamin1xb antibody. High-resolution confocal analyses revealed the presence of dynamin1xb in synatotagmin1- labeled presynaptic terminals that contact the cell bodies of motor neurons in the gray matter of the spinal cord **(B)**. Similarly, also in the visual cortex **(D)**, we observed a dynamin1xb immunolabeling signal that largely overlapped with synapses as judged by anti-synaptotagmin1 immunolabeling. Arrow in **(C)** points to an exemplary axon in the white matter of the spinal cord that was immunolabeled with anti-β-tubulin antibodies. **(A,C)** were obtained by epifluorescence microscopy; **(B,D)** by confocal microscopy. Abbreviations: n, nucleus of a motor neuron in the anterior horn of the spinal cord; mol, molecular layer; e-gran, external granule cell layer. Scale bars: 50 μm **(A,C)**; 10 μm **(B)**; 30 μm **(D)**.

**Figure 8:**
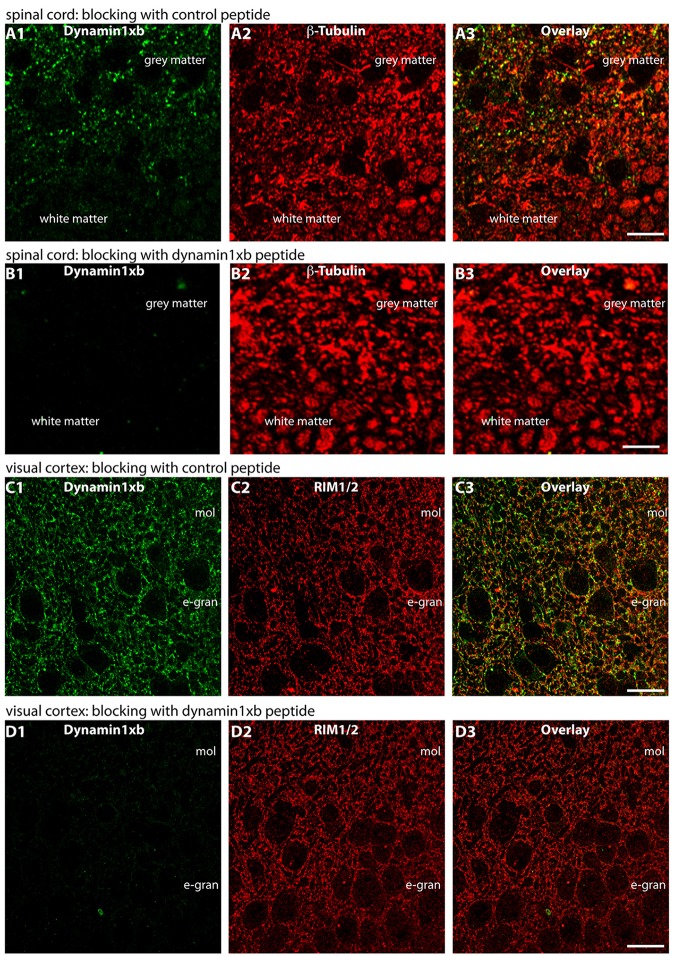
Semi-thin (0.5 μm-thin) sections of the mouse spinal cord **(A,B)** or mouse visual cortex **(C,D)** immunolabeled with the monoclonal antibody against dynamin1xb that was pre-absorbed either with a control peptide **(A,C)** or with the dynamin1xb peptide “PP12” against which the monoclonal antibody was raised **(B,D)**. The strong dynamin1xb imunosignal in the synaptic layers of the spinal cord and the visual cortex was completely abolished if the antibody against dynamin1xb was pre-absorbed with the “PP12” peptide **(B,D)** whereas the synaptic immunolabel of dynamin1xb was completely unaffected if a control peptide was used **(A,C).** Anti-β-tubulin immunolabeling in **(A,B)** and anti-RIM immunolabeling in **(C,D)** was completely unaffected by both of these treatments. Figure 8 was obtained by confocal microscopy. Abbreviations: mol, molecular layer; e-gran, external granule cell layer. Scale bars: 10 μm.

Also in the spinal cord, dynamin1xb was predominantly found in the gray matter that contains the bulk of synapses and to a much lesser amount in the white matter where fiber tracts predominate (Figures 7A,C). High resolution immunolabeling analyses revealed that dynamin1xb was enriched in presynaptic terminals in the gray matter of the spinal cord that were immunolabeled with anti-synaptotagmin1 (Figure 7B). Similarly, dynamin1xb immunolabeling of the visual cortex was also compatible with a synaptic distribution of dynamin1xb in that brain region (Figure 7D). Also in these experiments, the specificity of the immunolabeling results was further corroborated by pre-absorption experiments with the indicated peptides (Figure 8).

**Figure 9:**
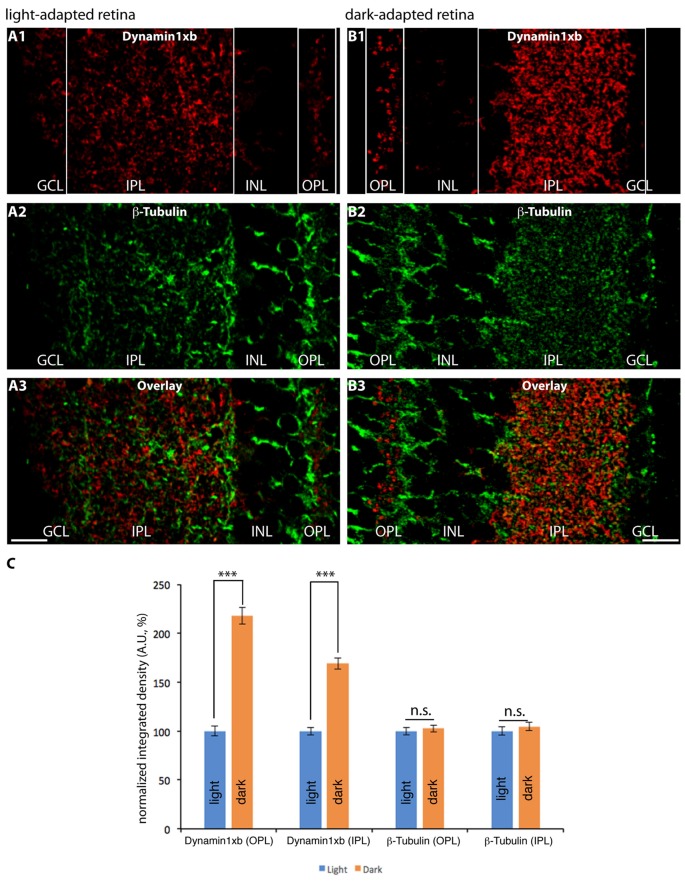
Semi-thin (1.5 μm-thin) sections of light- **(A)** and dark- **(B)** adapted retinas double-immunolabeled with antibodies against dynamin1xb **(A1,3**; **B1,3)** and β-tubulin **(A2,3**; **B2,3)**. As shown above (Figures 3, 4), the dynamin1xb immunosignal was enriched in the synaptic layers of the retina, the OPL and IPL. In the dark-adapted condition, we observed an enhanced dynamin1xb immunosignal in the synaptic layers of the OPL and IPL, while the β-tubulin immunosignal in the synaptic layers was unchanged. The boxed regions (white boxes) indicate the regions of interest, i.e., OPL and IPL, used for the quantification of immunofluorescence (IF) signals. **(A,B)** were obtained by confocal microscopy. **(C)** Quantification of the immunosignals in the OPL and IPL for dynamin1xb and β-tubulin (normalized data). Quantification of IF signals (detemined as integrated density) was done as previously described (Wahl et al., 2016). *N* = 3 embeddings for light- and dark-adapted retinas; *n* = 97 images analyzed for both light-and dark-adapted retinas. Error bars are SEM. Abbreviations: A.U, arbitrary units; OPL, outer plexiform layer; INL, inner nuclear layer; IPL, inner plexiform layer; GCL, ganglion cell layer. ***p < 0.001; n.s., non significant. Scale bars: 20 μm.

Since dynamin1xb is a Ca^2+^-regulated dynamin1 splice variant, we tested whether dynamin1xb is differentially distributed in synapses of light and dark-adapted retinas. Photoreceptor synapses in the outer retina are tonically active ribbon synapses with a particularly high basal synaptic vesicle turnover in the dark (Jackman et al., 2009). Therefore, we stained sections of light- and dark-adapted retinas with antibodies against dynamin1xb. Co-immunolabeling experiments of the same sections with antibodies against tubulin served as control incubations, e.g., to control differences in immunosignals due to possible minor differences in section thickness. The β-tubulin immunolabeling (reference immunostaining) shown in Figure 9 is very similar to previously published observations on the distribution of tubulin in the retina (e.g., Grayson et al., 2002). Identical conditions were applied for the experiments and for the subsequent analysis of the immunolabeled sections. The analyses were done blindly, i.e., without knowing whether the retina was from a light- or dark-adapted animal. In the dark-adapted samples, we observed an increased dynamin1xb immunolabeling particularly in the OPL. To a slightly lesser extent, also the IPL showed a stronger dynamin1xb immunolabel in the dark-adapted retinas in comparison to the light-adapted retinas. The β-tubulin immunosignal in the synaptic layers of the retina was indistinguishable between light- and dark-adapted retinas indicating that the differences in the synaptic immunolabeling intensity of dynamin1xb is not due to variations in section thickness or due to a global protein redistribution to synapses in the dark-adapted retinas.

## Discussion

In the present study, we analyzed the distribution of the activity-regulated dynamin1 splice variant dynamin1xb. Dynamin1xb is a unique splice variant because it contains a docking site for the Ca^2+^-/calmodulin-regulated phosphatase calcineurin that can switch-on the phosphorylation-dependent activities of dynamin1xb (Bodmer et al., 2011; Xue et al., 2011). In order to analyze the distribution of dynamin1xb, we used a splice-site selective monoclonal antibody that specifically detects a seven aa residues long peptide that is present only in dynamin1xb but not in dynamin1xa. Using this splice-site specific antibody against dynamin1xb, we found dynamin1xb enriched in synaptic regions in all brain regions that we analyzed. Therefore, dynamin1xb appears to fulfill a synaptic function that is needed in all types of synapses. High resolution analyses of retinal photoreceptor synapses demonstrated the enrichment of dynamin1xb in presynaptic terminals. Presynaptic terminals of brain synapses possess an intense vesicle traffic that is essential for synaptic communication (Südhof, 2004; Fernández-Alfonso and Ryan, 2006; Schweizer and Ryan, 2006; Soykan et al., 2016). Not only exocytotic but also endocytic pathways contribute to this process (Südhof, 2004; Schweizer and Ryan, 2006; LoGiudice and Matthews, 2007; Wu L.-G. et al., 2007; Dittman and Ryan, 2009; Yamashita, 2012; Rizzoli, 2014; Wu X.-S. et al., 2014; Kononenko and Haucke, 2015; Soykan et al., 2016; Watanabe and Boucrot, 2017. In presynaptic terminals, main function of endocytosis is the clearance of the active zone and the replenishment of synaptic vesicles (Hua et al., 2013; Rajappa et al., 2016; for review, see Yamashita, 2012; Kononenko and Haucke, 2015; Soykan et al., 2016; Watanabe and Boucrot, 2017). Dynamin1 is essential for synaptic vesicle endocytosis (Ferguson et al., 2007; for review, see Ferguson and De Camilli, 2012; Wu L.-G. et al., 2014; Cousin, 2015; Kononenko and Haucke, 2015; Soykan et al., 2016). Various types of endocytosis exist in presynaptic terminals that differ in functional properties, including speed/kinetics of membrane internalization/vesicle recycling, site of vesicle retrieval, timing and size of Ca^2+^ signals, temperature dependance, synaptic maturation, type of triggering and the underlying molecular mechanisms (Renden and von Gersdorff, 2007; Watanabe et al., 2013a,b; Midorikawa et al., 2014; Delvendahl et al., 2016; Soykan et al., 2017; for review, see Ferguson and De Camilli, 2012; Wu L.-G. et al., 2007, 2014; Yamashita, 2012; Cousin, 2015; Kononenko and Haucke, 2015; Gross and von Gersdorff, 2016; Soykan et al., 2016; Watanabe and Boucrot, 2017). Most of these different types of endocytosis, including the recently discovered ultrafast endocytosis (UFE; Watanabe et al., 2013a,b; Delvendahl et al., 2016; Soykan et al., 2017) and fast endophilin-mediated endocytosis (FEME; Boucrot et al., 2015; Renard et al., 2015), depend on dynamin1 (Ferguson et al., 2007; Pelassa et al., 2014; Wu X.-S. et al., 2014; Cousin, 2015; Soykan et al., 2016; Watanabe and Boucrot, 2017). In the presynaptic terminals, endocytosis is stimulated by synaptic activity (Ferguson et al., 2007; Hosoi et al., 2009; Wu et al., 2009; Wu X.-S. et al., 2014; Wu and Wu, 2014). The role of Ca^2+^ in different types of endocytosis is not completely understood and is partly controversially discussed (Wu et al., 2009; Yao et al., 2012; Wu X.-S. et al., 2014; but see von Gersdorff and Matthews, 1994; Leitz and Kavalali, 2011; for review, see Hosoi et al., 2009; Yamashita, 2012; Wu and Wu, 2014; Kononenko and Haucke, 2015; Gross and von Gersdorff, 2016). Particularly fast endocytosis appears to be stimulated by (transient) activity-dependent increases in Ca^2+^ (e.g., Neves et al., 2001; for review, see Hosoi et al., 2009; Wu et al., 2009; Yamashita, 2012; Wu L.-G. et al., 2014; Kononenko and Haucke, 2015).

Dynamin1xb could be particularly relevant for activity-regulated processes that depend on its interaction with syndapin. As outlined above, the dynamin1/syndapin interaction is phosphorylation-sensitive and inhibited by phosphorylation of distinct serine residues in the PRD of dynamin1 (Bodmer et al., 2011; Xue et al., 2011; Luo et al., 2016; for review, see Cousin, 2015). These serines are de-phosphorylated by the Ca^2+^-/calmodulin-stimulated phosphatase calcineurin thus promoting interaction between dynamin1 and syndapin (Anggono et al., 2006; for review, see Cousin, 2015). Remarkably, dephosphorylation of these serines in the PRD of dynamin1 leads to an activity-dependent acceleration of endocytosis in hippocampal neurons (Armbruster et al., 2013). Phospho-sensitive dynamin1–syndapin interaction is important for activity-dependent endocytosis that may occur as bulk endocytosis or other types of activity-dependent endocytosis (Anggono et al., 2006; Clayton et al., 2008, 2009, 2010; Wu X.-S. et al., 2014; for review, see Clayton and Cousin, 2009; Wu L.-G. et al., 2014; Cousin, 2015; Watanabe and Boucrot, 2017).

In neuroendocrine cells, dynamin1, Ca^2+^ as well as the Ca^2+^-regulated dynamin1-syndapin interaction have been reported to be essential for fusion pore expansion and in shifting the equilibrium between “kiss and run” exocytosis and “full-collapse” fusion (Elhamdani et al., 2001, 2006; Artalejo et al., 2002; Graham et al., 2002; Holroyd et al., 2002; Anantharam et al., 2011, 2012; Samasilp et al., 2012, 2014; Mattila et al., 2015; Zhao et al., 2016). Possibly, a similar function could apply for similar events in synapses of the central nervous system (Klingauf et al., 1998; Harata et al., 2006) and might be mediated by dynamin1xb.

We also found dynamin1xb enriched in the synaptic layers of the retina, the OPL and IPL, respectively. The OPL contains the tonically active photoreceptor ribbon synapses (Matthews and Fuchs, 2010). Photoreceptor synapses of the retina are large synapses with a single large active zone and therefore very suitable for high resolution immunohistochemical analyses (Wahl et al., 2013, 2016; Dembla et al., 2014). High resolution confocal microscopy revealed that dynamin1xb is localized close to the active zone in rod photoreceptor synapses. The dynamin1xb labeling pattern was similar to the previously described immunolabeling of the peri-active zone with a non-selective dynamin1 antibody (Wahl et al., 2013). In this study, dynamin1 was shown to be highly enriched at the peri-active zone, i.e., immediately lateral to the active zone, using immunogold electron microscopy (Wahl et al., 2013). Due to the close vicinity to the synaptic ribbon and the active zone (demonstrated in this study for photoreceptor ribbon synapses), dynamin1xb is a particularly promising candidate that could help to couple fast and transient increases of presynaptic Ca^2+^ into an activity-regulated endocytic membrane uptake. As mentioned above, dynamin1xb contains a unique docking site for the Ca^2+^-regulated phosphatase calcineurin (Bodmer et al., 2011; Xue et al., 2011). Calcineurin is highly enriched in the presynaptic terminals of photoreceptor synapses close to the synaptic ribbon (Wahl et al., 2013). Thus, calcineurin is available to dock onto dynamin1xb in the peri-active zone. If Ca^2+^ enters the presynaptic terminal e.g., via depolarization-induced opening of Cav-channels, dynamin1xb can be expected to be activated by Ca^2+^-regulated de-phosporylation of calcineurin and thus leading to compensatory endocytosis.

Interestingly, we observed an increased dynamin1xb immunosignal in the synaptic layers of dark-adapted retinas (in comparison to light-adapted retinas). The immunosignals for β-tubulin, that served as reference protein, were unchanged under these conditions. This light/dark difference was particularly strong in the OPL in which photoreceptor synapses are located. In this layer, photoreceptor synapses transmit the light stimuli for further processing to the inner retina. In darkness, photoreceptors possess a particularly active synaptic vesicle cycle (Jackman et al., 2009) with a high need for activity-dependent membrane retrieval. Therefore, the increased immunosignal in the OPL could indicate an activity-dependent recruitment and enrichment of dynamin1xb at the peri-active zone of photoreceptor presynaptic terminals in dark-adapted retinas.

Surprisingly, the IPL of dark-adapted retinas also showed a strongly increased dynamin1xb immunosignal. This increase of dynamin1xb in the IPL was highly significant and specific; the reference protein (β-tubulin) did not show a quantitative difference in immunolabeling intensity in the synaptic layers between light- and dark-adapted retinas. The IPL contains a mixed population of synapses that are either activated by light (“ON” synapses) or inactivated by light (“OFF” synapses) with different signaling properties (Tian, 2004; Lukasiewicz, 2005; Odermatt et al., 2012; Baden et al., 2014; Euler et al., 2014; Behrens et al., 2016; Franke et al., 2017; Real et al., 2017). If activity regulates synaptic recruitment of dynamin1xb also in the IPL, the darkness-induced increase of dynamin1xb in the IPL might be due to a particularly strong recruitment of dynamin1xb to OFF bipolar cell terminals (in comparison to ON bipolar terminals) and to other synapses in the IPL that are particularly active in the dark. Alternatively, the increased dynamin1xb immunosignal in the OPL and IPL of dark-adapted retinas could also result—at least in part—from an increased protein biosynthesis of dynamin1xb during the ≈4.5 h of dark adaptation. Previous studies did not observe an obvious difference in global protein synthesis in light- and dark-adapted retinas (Ames et al., 1980; Hollyfield and Anderson, 1982). But in some cases, an illumination-dependent enrichment of distinct proteins in retinal sub-compartments was observed that was either due to illumination-dependent protein synthesis (Iuvone and Beshearse, 1983; Hiragaki et al., 2014; Hughes et al., 2015; Wolloschek et al., 2015; Vancura et al., 2016) or based on an endogenous circadian rhythm (Tosini and Menaker, 1996; Tosini et al., 2007; Wolloschek et al., 2015; Vancura et al., 2016). Furthermore, light-dependent translocation might play a role. Light-dependent subcellular translocation is well known for proteins of the visual transduction cascade in photoreceptors, e.g., transducin, arrestin, unc119a (Whelan and McGinnis, 1988; Artemyev, 2008; Kerov and Artemyev, 2011; Majumder et al., 2013; Sinha et al., 2013). It is unlikely that the circadian rhythm plays a major role for the observed changes in dynamin1xb accumulation in the synaptic layers because light- and dark-adaptation were done simultaneously, i.e., at the same time in the afternoon. But clearly, future analyses are required to further discriminate between these possibilities. For these experiments and the further characterization of dynamin1 splice variants, the dynamin1xb-specific monoclonal antibody will be a useful tool. Calcineurin, the interaction partner of dynamin1xb, could be an important mediator of the activity-regulated adaptation of the endocytic machinery in retinal synapses. Calcineurin dephosphorylates NFAT proteins that in turn translocate to the nucleus to regulate gene transcription in various systems (for review, see Crabtree and Olson, 2002; Wu H. et al., 2007). Similar mechanisms might contribute to the observed synaptic changes of dynamin1xb in dark-adapted retinal synapses. Recently, calcineurin was shown to be an essential mediator of homeostatic synaptic plasticity that serves to adjust synapses and neuronal circuits to different levels of network activity (Arendt et al., 2015). Such a regulatory homeostatic regulation is particularly needed in the retina because the retina efficiently operates at very different levels of environmental light intensities (for review, see Dunn and Rieke, 2006; Rieke and Rudd, 2009; Gollisch and Meister, 2010; Lagnado and Schmitz, 2015). Our observations demonstrate an illumination-dependent remodeling of dynamin1xb at the mouse photoreceptor ribbon synapse that most likely affects endocytic vesicle trafficking in the peri-active zone. Interestingly, in drosophila, an illumination-dependent reorganization also of the active zone of photoreceptor synapses was described (Böhme and Sigrist, 2015; Sugie et al., 2015). The detailed molecular mechanisms of these adaptative processes in retinal synapses have to be addressed by future investigations.

## Author Contributions

M-LE designed and conducted embedding, western blot and imaging experiments, analyzed data and wrote the manuscript together with ED and FS. ED designed and conducted embedding and imaging experiments, analyzed data and wrote the manuscript together with M-LE and FS. SW conducted western blot and dot blot experiments and analyzed data. MD conducted tissue embedding and imaging experiments and analyzed data. KS conducted embedding and imaging experiments and analyzed data. FS designed and supervised the study, analyzed data and wrote the article together with M-LE and ED.

## Conflict of Interest Statement

The authors declare that the research was conducted in the absence of any commercial or financial relationships that could be construed as a potential conflict of interest.
